# DNA methylation of SPARC and chronic low back pain

**DOI:** 10.1186/1744-8069-7-65

**Published:** 2011-08-25

**Authors:** Maral Tajerian, Sebastian Alvarado, Magali Millecamps, Thomas Dashwood, Kathleen M Anderson, Lisbet Haglund, Jean Ouellet, Moshe Szyf, Laura S Stone

**Affiliations:** 1 Alan Edwards Centre for Research on Pain, McGill University, 740 Dr. Penfield Avenue, Montreal, Quebec, H3A 1A4, Canada; 2 McGill Scoliosis & Spine Research Group, McGill University Health Centre, 1650 Cedar Avenue, Montreal, Quebec, H3G 1A4, Canada; 3 Department of Neurology and Neurosurgery, McGill University, Faculty of Medicine, 3801 University Street, Montreal, Quebec, H3A 2B4, Canada; 4 Department of Pharmacology and Therapeutics, McGill University, Faculty of Medicine, 3655 Promenade Sir William Osler, Montréal, Québec, H3G 1Y6, Canada; 5 Sackler Program for Epigenetics & Developmental Psychobiology, McGill University, 3655 Promenade Sir William Osler, Montreal, Québec H3G 1Y6, Canada; 6 Faculty of Dentistry, McGill University, 3640 University Street, Montreal, Quebec, H3A 2B2, Canada; 7 Department of Physical Medicine & Rehabilitation, University of Minnesota Medical School, 420 Delaware Street S.E., Minneapolis, MN, 55454, USA; 8 Orthopaedics Research Laboratory, McGill University Health Centre, 687 Pine Avenue West, Montreal Quebec, H3A 1A1, Canada; 9 Division of Orthopaedic Surgery, McGill University Health Centre, 1650 Cedar Avenue, Montreal, Quebec, H3G 1A4, Canada; 10 Department of Anesthesiology, Anesthesia Research Unit, McGill University, Faculty of Medicine, 3655 Promenade Sir William Osler, Montreal, Quebec, H3G 1Y6, Canada

**Keywords:** SPARC, back pain, DNA methylation, epigenetics, intervertebral disc, aging, gene expression, disc degeneration

## Abstract

**Background:**

The extracellular matrix protein SPARC (Secreted Protein, Acidic, Rich in Cysteine) has been linked to degeneration of the intervertebral discs and chronic low back pain (LBP). In humans, SPARC protein expression is decreased as a function of age and disc degeneration. In mice, inactivation of the SPARC gene results in the development of accelerated age-dependent disc degeneration concurrent with age-dependent behavioral signs of chronic LBP.

DNA methylation is the covalent modification of DNA by addition of methyl moieties to cytosines in DNA. DNA methylation plays an important role in programming of gene expression, including in the dynamic regulation of changes in gene expression in response to aging and environmental signals.

We tested the hypothesis that DNA methylation down-regulates SPARC expression in chronic LBP in pre-clinical models and in patients with chronic LBP.

**Results:**

Our data shows that aging mice develop anatomical and behavioral signs of disc degeneration and back pain, decreased SPARC expression and increased methylation of the SPARC promoter. In parallel, we show that human subjects with back pain exhibit signs of disc degeneration and increased methylation of the SPARC promoter. Methylation of either the human or mouse SPARC promoter silences its activity in transient transfection assays.

**Conclusions:**

This study provides the first evidence that DNA methylation of a single gene plays a role in chronic pain in humans and animal models. This has important implications for understanding the mechanisms involved in chronic pain and for pain therapy.

## Background

Chronic low back pain (LBP) is a complex continuum of painful conditions that includes both axial and radicular pain [1]: Axial LBP is defined as spontaneous or movement-evoked pain or discomfort localized to the spine and low back region. Non-axial, radiating LBP is pain in one or both legs. Often referred to as radicular pain or sciatica, it usually follows the course of the sciatic nerve. Current diagnostic and therapeutic approaches to chronic back pain are limited by our narrow understanding of the underlying biological mechanisms. There are many potential causes of chronic LBP including degenerative disc disease (DDD). While natural age-related degeneration of intervertebral discs (IVDs) is common [2,3], chronic LBP is associated with increased signs of disc degeneration [4,5]. Like most other conditions, back pain is the product of genetic [6,7] and environmental [8,9] influences.

SPARC (secreted protein, acidic, rich in cysteine; aka osteonectin or BM-40) is an evolutionarily conserved collagen-binding protein present in IVDs. SPARC is known to influence bone remodeling, collagen fibrillogenesis, and wound repair [10]. Decreased expression of SPARC has been associated with aging and degeneration in human IVDs [11]. Furthermore, targeted deletion of the SPARC gene results in accelerated disc degeneration in the aging mouse and a behavioral phenotype resembling chronic LBP in humans [12,13]. The genetic evidence from mice and the clinical observation that SPARC is down-regulated in humans with disc degeneration suggests that long-term down-regulation of SPARC expression may play a critical role in chronic LBP. What are the mechanisms that could lead to lasting down-regulation of genes such as SPARC?

One mechanism that is now well established for stable, long-term programming of gene expression is DNA methylation. The DNA is covalently modified by the addition of methyl moieties by an enzymatic DNA methyltransferase reaction that catalyzes the transfer of a methyl group from the methyl donor S-adenosyl methionine. What distinguishes DNA methylation in vertebrate genomes is the fact that not all CpGs are methylated in any given cell type, generating cell type-specific patterns of methylation [14], which confer upon a genome its cell type-specific identity. Active regulatory regions of the chromatin, which enable gene expression, are associated with hypomethylated DNA, whereas hypermethylated DNA is packaged in inactive chromatin resulting in gene silencing [15,16]. Patterns of DNA methylation are generated during gestation and until recently were believed to be restricted to life-long programming of cell type-specific gene expression [17]. However, recent data suggests that DNA methylation is dynamic in adult non-dividing cells and is responsive to environmental signals [18]. It might therefore play a role in the modulation of gene function in response to a plethora of environmental signals after birth and throughout life [19].

We tested the hypothesis that DNA methylation occurring later in life or in pathological conditions might play a role in chronic LBP. The possibility that DNA methylation might precipitate chronic pain through down-regulation of expression of critical genes such as SPARC has not been previously addressed.

Our results are consistent with the hypothesis that alterations in DNA methylation associate with chronic LBP and IVD degeneration in mice and in humans with chronic LBP. This data provides the first line of evidence that supports the hypothesis that DNA methylation is involved in chronic pain.

## Results

### Aging and SPARC-null mice exhibit signs of disc degeneration and back pain

**Figure 1:**
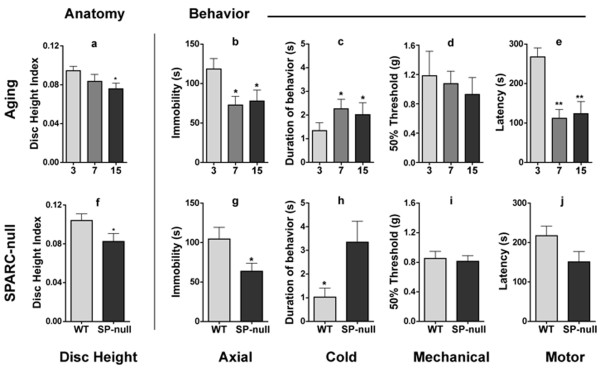
**Disc degeneration and behavioral signs of low back pain in aging and SPARC-null mice**. *Anatomy: *The disc height index measured in 15-month old mice is smaller than that in 3-month old mice (a). 4-month old SPARC-null mice show smaller average disc height compared to age-matched WT controls (f). *Pain Behavior: *Aging mice exhibit signs of axial discomfort (b), cold sensitivity (c), but not mechanical sensitivity (d) in the hindpaw, in addition to overall motor impairment (e) when compared to 3-month old mice. 4-month old SPARC-null animals show a behavioral profile similar to older WT mice (g-j). * = p < 0.05, ** = p < 0.01, One-way ANOVA followed by Bonferroni's test (aging cohorts), two-tailed student t-test (SPARC-null vs WT). n = 8-15/group. Error bars = S.E.M.

Disc degeneration is known to increase as a function of aging in both humans and rats [3,20] and is associated with an increased risk of chronic LBP [4]. In order to determine the effect of aging on disc degeneration in mice, disc height was determined from x-rays of the lumbar spine in young (3 month), middle-aged (7 month), and old (15 month) C57B wild-type (WT) mice. Our data shows reduction in lumbar disc height as a function of aging (Figure 1a). A similar decrease in disc height was observed in young 4-month old SPARC-null mice compared to their age-matched WT controls, such that a 4-month old SPARC-null mouse is roughly equivalent to a normal 15-month old animal in terms of disc anatomy (Figure 1f). Thus, both normal aging and the deletion of the SPARC gene results in disc degeneration in mice, supporting a role for SPARC in maintaining disc integrity.

We then tested whether age-dependent disc degeneration in mice is associated with behavioral signs of axial and radicular pain and motor impairment, and whether it is impacted by loss of function of SPARC.

Axial pain was assessed using a modified version of the tail suspension assay in which the spontaneous reaction to gravity-induced strain along the axis of the spine was measured [21]. Young animals (3 months) spent significantly more time in the immobility posture than did older animals (Figure 1b). This implies a reluctance to stretch the body axis, which is suggestive of axial pain. A similar decrease in immobility was observed in 4-month old SPARC-null mice compared to their age-matched WT controls (Figure 1g).

Sensitivity to mechanical and cold stimuli in the hindpaw were used as behavioral indices of radicular pain. While 7- and 15-month old WT mice developed hypersensitivity to cold as a function of age (Figure 1c), mechanical paw withdrawal thresholds were not significantly altered (Figure 1d). Similarly, 4-month old SPARC-null mice presented with cold but not mechanical hypersensitivity compared to their WT controls (Figure 1h,i).

Physical ability was assessed using the accelerating rotarod assay, in which the latency to fall from a rotating treadmill is measured. Aging WT mice showed impaired performance on this task at 7- and 15-months of age, suggesting the presence of movement-evoked pain and/or motor dysfunction (Figure 1e). SPARC-null animals showed a trend towards impaired motor function compared to WT mice at 4-months of age (Figure 1j).

Together, these data indicate that naive WT mice develop age-dependent disc degeneration along with behavioral signs of axial and radicular low back pain and motor impairment.

### Aging mice demonstrate changes in expression and DNA methylation of SPARC

**Figure 2:**
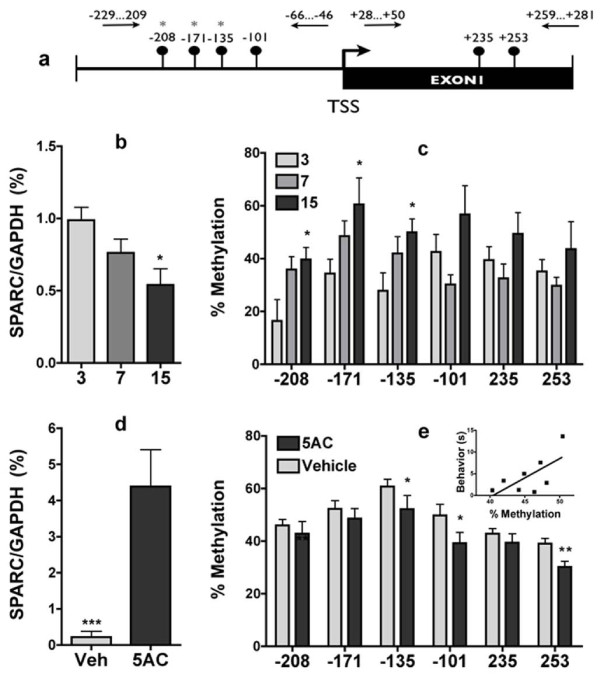
**Changes in expression and DNA methylation of SPARC in aging mice**. SPARC mRNA expression (relative to *gapdh*) at different time points in life (b). Age-dependent changes in methylation of CG sites in the SPARC promoter (a) in IVDs as quantified by pyrosequencing (c). Treatment of 1-year old mice with the demethylating drug 5AC or vehicle (30 mg/kg, i.v. and 250 fmol i.t.) resulted in a 4-fold increase in SPARC mRNA expression and (d) decreased methylation of CG sites in the SPARC promoter as quantified by pyrosequencing in IVDs. Inset: Increased cold sensitivity following 5AC injection was significantly correlated with total SPARC methylation. * = p < 0.05, *** = p < 0.001. One-way ANOVA followed by Bonferroni's test (aging cohorts) and two-tailed student t-test (5AC vs. vehicle treated). Pearson's correlation (p = 0.03, r^2 ^= 0.46). n = 3-15/group. Error bars = S.E.M.

The aging phenotype in WT mice is remarkably similar to 4-month old SPARC-null mice, suggesting that the absence of the SPARC gene accelerates the normal aging process in the IVDs, resulting in low back pain. We therefore tested whether aging is accompanied by reduced SPARC expression. A significant decrease in SPARC mRNA expression was observed in lumbar IVDs taken from 15-month-old mice compared to 3-month-old mice (Figure 2b). This is consistent with the hypothesis that age-dependent down-regulation of SPARC might be associated with chronic LBP and that mechanisms that stably down-regulate SPARC expression are involved in precipitating chronic pain.

We therefore addressed the question of what is the mechanism that down-regulates SPARC during aging. The SPARC promoter has been shown previously to be modulated by DNA methylation in cancer. Specifically, it is hypermethylated and silenced in the majority of invasive cervical cancer cases [22]. Cancer epigenetics has taught us that tumor suppressor genes that are suppressed in cancer because of genetic mutations are silenced in many other cases by DNA methylation, leading to similar phenotypes. Interestingly, genes that are frequently hypermethylated in cancer are also hypermethyled with age [23]. We therefore hypothesized that a similar mechanism operates in chronic LBP, whereby the age-dependent decrease in SPARC mRNA expression is due to increased methylation of the SPARC promoter. DNA was prepared from mouse lumbar discs and the state of methylation of the SPARC promoter region was mapped in 3-, 7-, and 15-month old mice. A pyrosequencing analysis of the methylation state of 6 CpG sites residing in the SPARC gene promoter region (Figure 2a) showed increased methylation in IVDs with increasing age at several sites located in the promoter (Figure 2c).

### The DNA demethylating agent 5-azacytidine silences SPARC gene expression in the mouse IVD *in vivo*

To test whether DNA methylation is involved in silencing SPARC gene expression in IVD *in vivo*, we treated 1-year old mice with the DNA demethylating agent 5-azacytidine (5AC) (30 mg/kg, i.v. and 250 fmol i.t.). We observed a significant > 4-fold increase in SPARC mRNA in the 5AC-treated mice compared to the vehicle-treated controls (Figure 2d). A pyrosequencing analysis revealed demethylation of the SPARC promoter in animals treated with 5-AC (Figure 2e). To further test the involvement of SPARC methylation in chronic pain we determined the relationship between state of methylation of the SPARC promoter and cold allodynia as a measure of pain. Our data indicates that the state of DNA methylation was significantly correlated with the magnitude of cold allodynia *in vivo *(Figure 2e, inset).

### The mouse and human SPARC promoters are silenced by DNA methylation *in vitro*

**Figure 3:**
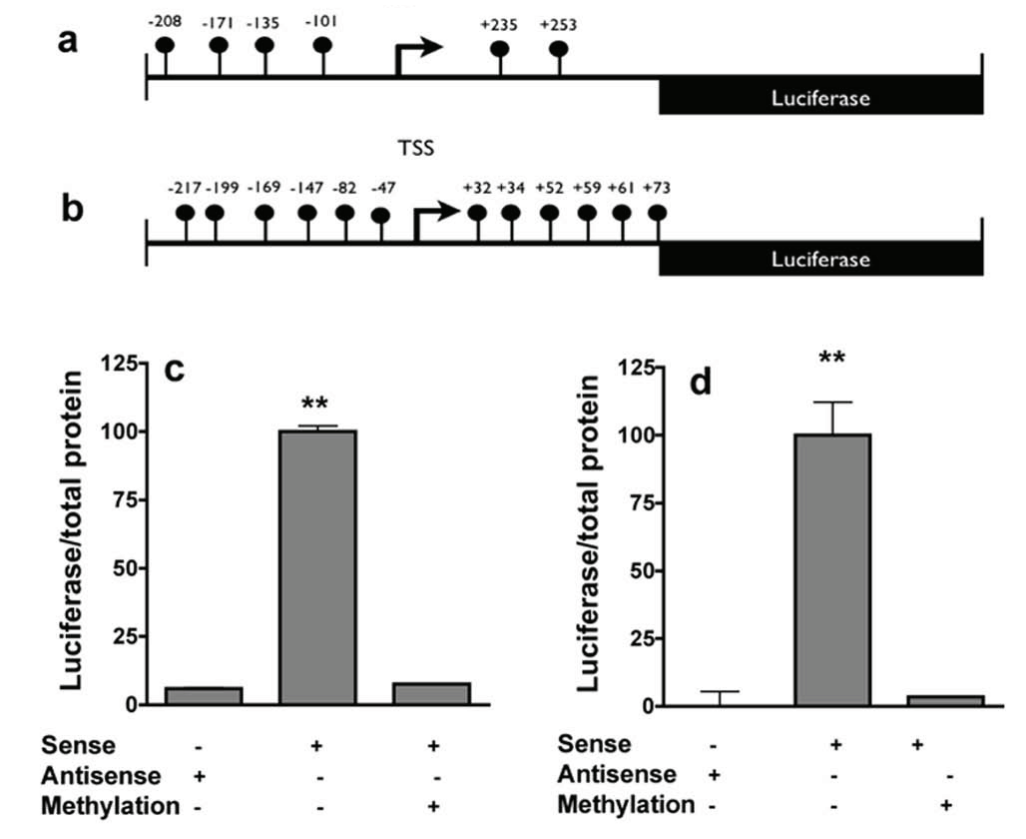
**DNA methylation silences murine and human SPARC promoter activity**. The mouse (a) and human promoter regions (b) were cloned into a pCPGL-basic plasmid (the CpG positions are indicated as balloons). There were no CpGs in the vector. The plasmids were either methylated or mock methylated *in vitro *and then transfected into HEK293 cells in the sense or antisense (1^st ^column) configuration for 48 h (c, d). Relative luciferase activity (percentage) in the extracts is shown as average per group (triplicate transfection). The decrease in expression in the methylated (3^rd ^column) vs. unmethylated (2^nd ^column) treatment groups indicates gene silencing. One-way ANOVA followed by Bonferroni's test. ** = p < 0.01 Error bars = S.E.M.

Our results with 5AC presented in Figure 2 show that non-specific demethylation can activate the SPARC gene *in vivo*. In order to directly determine whether methylation of the SPARC gene promoter region silences either mouse or human SPARC promoters we subcloned the promoter region into a CpG-deficient luciferase reporter plasmid pCpGL [24]. The same region was subcloned in the antisense direction as a negative control. We methylated exclusively the sites that were shown to be methylated *in vivo *using Sss1 DNA methyltransferase (Figure 3a, b), and compared luciferase activity after transient transfection into HEK293 cells with the same plasmid that was mock-methylated. The luciferase expression observed in the control condition was completely silenced by methylation of both the mouse (Figure 3c) and human SPARC promoters (Figure 3d), demonstrating that methylation silences SPARC promoter activity.

### SPARC promoter is hypermethylated in LBP patients with disc degeneration

**Figure 4:**
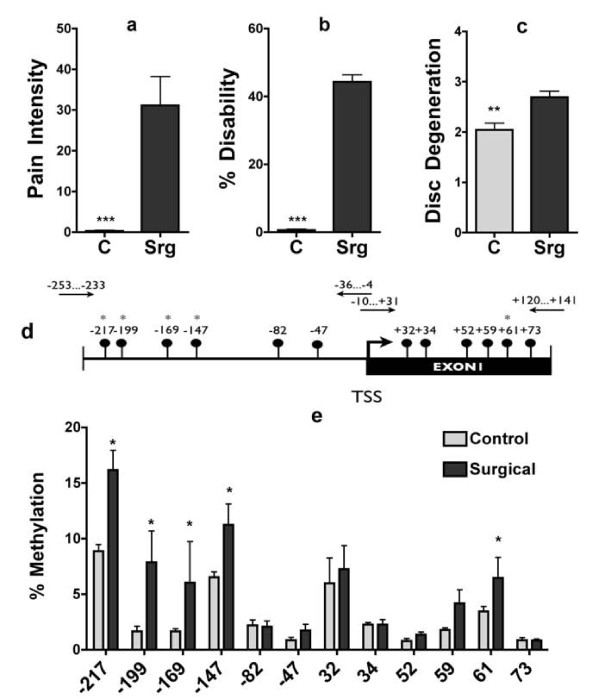
**SPARC mRNA expression and DNA methylation in IVDs from chronic LBP patients with disc degeneration**. Pain intensity measured with the numeric rating scale (a) and physical disability as determined by the Oswestry Disability Index (b) were increased in surgical patients, as was lumbar disc degeneration based on scoring of MRI images (c). The state of methylation of CG sites in the human SPARC promoter (d) in L4-L5 IVDs was increased as quantified by pyrosequencing (e) * = p < 0.05, ** = p < 0.01, *** = p < 0.001. Two-tailed student t-test. n = 5-8/group. Error bars = S.E.M.

Given the potential clinical significance of these findings, we examined whether these results could be translated to humans. Patients with severe chronic LBP were recruited from a pool of individuals scheduled for spinal fusion surgery due to severe disc degeneration. Pain free controls were recruited from the general population. As anticipated, the surgical group had higher pain and disability levels than control subjects (Figure 4a, b). The degree of degeneration of the lumbar spine was determined by lumbar MRI and was also significantly increased in patients vs. controls (Figure 4c). These data indicate that our patient population had significant increases in disc degeneration, pain, and disability compared to the general population.

Decreases in SPARC expression with age and degeneration have been reported elsewhere [11]. Unfortunately, the quality of the RNA derived from the human IVD didn't allow us to accurately measure SPARC mRNA. However, the stability of DNA allowed us to perform bisulfite mapping of the promoter region. Increased methylation of the SPARC promoter (Figure 4d) was observed in surgical samples compared to controls. Five out of the thirteen individual sites examined had significantly increased methylation in the surgical samples (Figure 4e). Our data supports the hypothesis that increased DNA methylation of promoters such as SPARC gene promoter are associated with pain in humans.

## Discussion

The prevalence of chronic pain in the general population is estimated at 15-40%. In addition to being a major health care problem, chronic pain has serious economic consequences, costing billions of dollars per year in lost productivity and medical expenses. Chronic pain is often resistant to therapeutic intervention, and individuals suffer for years without relief. New therapeutic strategies are desperately needed. Despite enormous efforts, significant advances in pain management continue to be elusive.

While a genetic basis for individual variations in the development of chronic pain is well established [25], genetics only accounts for approximately half of the inter-individual variability in chronic low back pain [26]. One concept that is beginning to receive attention in pain research is the fact that gene function can be altered not only by differences in gene sequence but also by differences in epigenetic modification. Epigenetic modulation refers to chemical modifications of DNA including DNA methylation that produce long-term changes in gene expression. These changes can have long-lasting biological consequences and could become maladaptive, leading to chronic diseases such as obesity [27], fatigue [28] or neurological and mental disorders [19,29].

In humans, decreased expression of SPARC is observed in painful, degenerating discs [11] and deletion of the SPARC gene triggers accelerated age-dependent disc degeneration [12] and chronic pain in mice [13]. We therefore hypothesized that the SPARC gene may be silenced by DNA methylation as a function of aging in degenerating discs.

In the current study we show that disc degeneration is accompanied by signs of axial and radicular pain and physical impairment in mice (Figure 1) and with pain and physical disability in humans (Figure 4). Targeted inactivation of the SPARC gene results in early onset of both disc degeneration and behavioral indices of LBP in mice [12,13]. Having established that deletion of SPARC increases chronic pain and disc generation, we determined whether this gene is commonly silenced by DNA methylation during normal aging. Increased disc degeneration and chronic pain are associated with age in rodents and humans.

There are multiple mechanisms by which IVD degeneration can result in chronic axial and/or radicular LBP. In the periphery, spinal instability due to disc degeneration could result in irritation of other structures such as the facet joints, muscles and ligaments. Increased innervation of degenerating discs by sensory neurons [30] is also thought to contribute to discogenic pain [31] and contact with the contents of the disc results in increased neuronal excitability and sensitization [32,33]. In addition, disc degeneration may result in radicular pain following nerve compression due to disc bulging or herniation [34,35].

Within the central nervous system, ongoing nociceptive input from peripheral structures may result in sensitization within the spinal cord or supraspinal structures, resulting in an exaggerated response to subsequent peripherally applied stimuli (for reviews see [36,37]). Furthermore, chronic LBP results in changes in brain structure and function [38,39]. Interestingly, therapeutic interventions that target the spinal column such as facet joint blocks or spinal surgery can reverse pain-related changes in the brain, suggesting that ongoing input from the periphery actively maintains pain-related CNS plasticity [39]. While the current study is focused on the epigenetic modulation of the degeneration of a peripheral structure, studies examining the role of epigenetics in pain-related CNS plasticity are needed.

We provide several lines of evidence that support the hypothesis that DNA methylation occurs during aging and that it results in silencing of SPARC. First, the state of methylation of several CG sites in the promoter is increased with aging. Second, DNA methylation inhibitors resulted in demethylation of the SPARC promoter and increased expression of SPARC *in vivo*. Third, methylation of CG sites in the promoter lead to silencing of promoter activity. The combination of the genetic evidence for the role of SPARC in chronic pain and the DNA methylation analysis provide strong support for the idea that DNA methylation occurs during aging and that it is involved in disc degeneration and chronic LBP. Although it was not possible to provide direct evidence of SPARC mRNA expression in clinical samples, we tested whether the clinical situation is consistent with our hypothesis. We show that discs removed from patients that suffered from chronic LBP also exhibited increased methylation of the SPARC gene (Figure 4). The human promoter, like the mouse promoter, is silenced by DNA methylation (Figure 3).

Unlike the static genome, the epigenome is in dynamic equilibrium throughout our lifespan and DNA methylation is a bidirectional process that can be altered by pharmacological agents [40]. Changes in methylation appear to be a common feature in aging cells and tissues. Epigenetics also plays a key role in the development of diseases associated with aging including cancer [41,42], atherosclerosis, and neurodegenerative and autoimmune disorders [43]. Interestingly, age itself is a risk factor for chronic pain in humans [44]. Using the candidate gene approach, we demonstrate that aging is one physiological process that could lead to hypermethylation and silencing of SPARC. It is likely that aging results in silencing of additional genes that are involved in chronic pain either in the periphery or the central nervous system. Moreover, it stands to reason that other transient environmental exposures such as tissue injury could result in DNA methylation of many other genes, which could serve as a long-term memory of such exposures in the genome, resulting in chronic pain.

## Conclusions

Our study provides the first line of evidence that DNA methylation is involved in chronic pain. Specifically, we present evidence from both mouse and human studies supporting the hypothesis that DNA methylation of the SPARC promoter is increased with age and intervertebral disc degeneration, resulting in the silencing of a gene that is protective against accelerated disc degeneration. The SPARC gene is likely to be just one example of many pain-relevant genes that are similarly regulated by DNA methylation in both peripheral tissues and in the central nervous system.

Epigenetic modifications are at the interface between environment and genetics, creating a mechanism by which life experience can lead to long-lasting changes in gene expression. If DNA methylation is implicated in chronic pain, it will provide not only new understanding of the underlying mechanisms involved in generation and maintenance of chronic pain, but also new therapeutic possibilities.

## Methods

### Animals

All procedures were approved by the Animal Care Committee at McGill University, and conformed to ethical guidelines of the Canadian Council on Animal Care. Female C57BL/6 mice were used in this study. The SPARC-null mouse [45] was backcrossed onto a standard C57BL/6 background for > 12 generations [46]. All animals were bred and aged in-house and experienced identical environments. Experiments were performed blind to genotype and age.

### Mouse Behavioral Assays

#### Axial Pain: Tail Suspension Assay

Mice were suspended underneath a platform by the tail with adhesive tape attached 0.5-1.0 cm from the base of the tail and videotaped for 3 minutes. The duration of time spent in a) immobility (not moving but stretched out), b) rearing (trying to reach the underside of the platform), c) full extension (actively reaching for the floor), and d) self-supported (holding either the base of its tail or the tape) were determined [21].

#### Radicular Pain: Sensitivity to Cold Stimuli

Spontaneous nociceptive behaviors (flinching, licking or biting) were measured for 1 minute after a drop of acetone (~25 μl) was applied to the hindpaw as previously described [13].

#### Radicular Pain: Sensitivity to Mechanical Stimuli

A series of calibrated monofilaments (von Frey filaments) were applied with increasing force to the plantar surface of the hindpaw until the animal responded by withdrawing. A decrease in threshold suggests the development of mechanical hypersenstivity. Results are expressed as the threshold (in grams) to withdraw 50% of the time as previously described [13].

#### Rotarod Assay

We used the accelerating rotarod assay to monitor animals for decreased motor function [47]. The latency to fall was recorded in seconds. The maximum duration of the test was 5 minutes.

### Radiographic assessment of IVD degeneration in Mice

**Figure 5:**
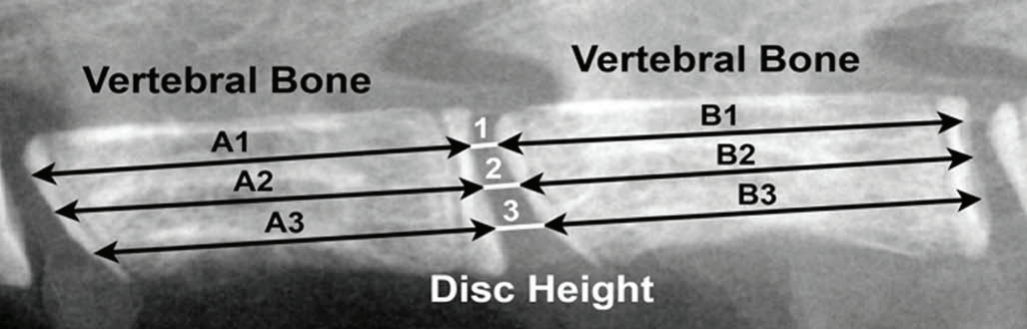
**Calculation of Disc Height Index from Mouse Lumbar Spinal X-ray**. Lateral x-ray images of the intact lumbar spine were taken at 4× using a Faxitron ^® ^MX-20 (Faxitron X-Ray LLC, Lincolnshire, IL). Disc Height Index (DHI) was determined according to the following equation: Disc Height Index (DHI) = 2 × (DH1 + DH2 + DH3)/(A1 + A2 + A3 + B1 + B2 + B3) where A and B represent the length of the vertebral bone immediately rostral and caudal to the IVD, respectively; and DH represents the disc height between adjacent vertebrae.

Aging WT mice were anaesthetized and x-rays were obtained using a Faxitron 3000. For SPARC-null and WT control mice, radiography was performed *ex vivo*. Disc height was calculated for all lumbar IVDs (Figure 5). The observer was blind to age and genotype.

### Human Subjects

Procedures involving human subjects were approved, as applicable, by the following Institutional Review Boards: McGill University Faculty of Medicine, McGill University Health Centre, The University of Minnesota and Allina Health Care (Minneapolis, MN).

Three groups of human subjects were used in this study. Additional information on human subjects can be found in Additional File 1.

#### Experimental Subjects

The experimental group was recruited from a pool of patients scheduled for spinal fusion to treat severe cLBP associated with lumbar disc degeneration (mean age = 45.6 ± 2.8, n = 10, 3 males, 5 females, 2 unknown; see Additional File 1). Following surgical removal, discs were rinsed briefly in PBS, sectioned into quadrants, flash frozen in liquid nitrogen and stored at -80C.

#### Healthy Pain-Free Controls

A healthy control group consisting of individuals free of back pain was used as a comparator for the pain and disability assessments and disc degeneration scores in the surgical samples. (mean age = 41.2 ± 2.3, n = 23, 14 males and 9 females). No tissue was available from these subjects.

#### Non-Degenerated Control Discs

Non-degenerated control IVDs were acquired with the cooperation of Transplant Quebec from tissue donors. Lumbar spinal columns were extracted intact and placed on ice for transport. While age, sex and cause of death is recorded, no information is available regarding the presence of back pain or physical disability before death. X-rays were obtained of lumbar spines prior to IVD dissection and discs with obvious signs of degeneration (reduced height, calcification, bony spurs) were excluded from the study. Following x-ray of the entire column, the IVDs were dissected and stored at -80 until use. (mean age = 58.2 ± 4.4, n = 5 males, see Additional File 1).

### Pain and disability assessment in human subjects

Pain was assessed using the numeric rating scale in which subjects rate pain intensity on a scale of 0-100, where 100 is the worst pain imaginable. Pain-related disability was determined using the Oswestry Disability Index (ODI) questionnaire [48].

### Radiographic assessment of IVD degeneration in human subjects

Lumbar MRIs were obtained for all surgical and healthy, pain-free control subjects. Each lumbar disc was scored using the 5-point Pfirrmann scale [49] by a radiologist blind to diagnosis.

### Bisulfite mapping and expression analyses

DNA was treated with sodium bisulfite and primers were designed for converted products of both mouse [GenBank:AL596207.10] and human [GenBank: AC011374.6] SPARC promoters. PCR products were sequenced using the Biotage Pyrosequencer according to the manufacturer's protocol (See Additional File 2 for primer sequences) [50]. Expression of *SPARC *[GenBank: AK003162.1] was quantified using quantitative RT-PCR on the Lightcycler 480 using *GAPDH *[GenBank: AK002273.1] for normalization.

RNA extraction was carried out using Trizol (Invitrogen) and followed by Dnase I treatment and cDNA conversion using random hexamers (Roche Molecular Biochemicals) according to manufacturer's instructions. Expression of SPARC was then quantified using quantitative RT-PCR on the Lightcycler 480 using GAPDH for normalization. *SPARC *primers for human and mouse are listed in Additional File 2.

Bisulfite PCRs were amplified using two rounds of PCR using outer and nested primers (see Additional File 2). Cycling conditions involved an initial step of 5 minutes at 95°C followed by 35 cycles of [95°C for 1 minute, T_m _for 2.5 minutes, 72°C 1 minute] Followed by 5 minutes of 72°C. Luciferase construct PCRs were made with primers (See Additional File 2) using the same cycling conditions only with one round of PCR. Quantitative PCR was amplified with a pre-incubation at 95°C for 10 minutes followed by 45 cycles of [95°C for 10 seconds, 60°C for 10 seconds, 72°C for 10 sec] followed by 10 minutes of 72°C.

### Luciferase reporter assay

Human and mouse SPARC promoters were subcloned into the CpG-less pCpGL luciferase reporter plasmid [24] in both 5' to 3' (sense) or 3' to 5' (antisense) orientation, respectively.

The constructs were methylated in vitro with SssI CpG DNA methyltransferases (New England Biolabs, Inc.) as recommended by the manufacturers.

Transfections were performed using calcium phosphate precipitation as described previously) [51]. Cells were harvested 48 h after transfection and luciferase activity was assayed using the Luciferase Assay System (Promega).

### Statistical analysis

All data are plotted as mean ± SEM. The student t-test (2-tailed, unpaired) was used in data comparing two groups. One-way ANOVA followed by Bonferroni's test was used when comparing 3 groups. For the luciferase assay, data is shown in the percentile form. All data was analyzed and graphed using Prism 4.0 (GraphPad Software, Inc., La Jolla, CA).

## List of Abbreviations

**SPARC**: Secreted Protein, Acidic, Rich in Cysteine; **WT**: wild-type; **LBP**: low back pain; **IVD**: intervertebral disc

## Competing interests

The authors declare that they have no competing interests.

## Authors' contributions

MT and SA Overall experimental design, collected the mouse behavioral data, performed DNA and RNA data collection and analysis and drafted the manuscript. MM developed the SPARC-null mouse model and established the methods for the collection of the behavioral data and the mouse IVDs. TD Contributed to DNA and RNA data collection and analysis. KMA Contributed to the identification, recruitment, diagnosis of the surgical patients and pain-free healthy controls and collected the pain and disability data. LH & JO Collection of human discs and assessment of disc degeneration. MS Overall design of the DNA methylation experiments, supervision of the experiments and writing of the manuscript. LSS Overall design of the *in vivo *human and mouse experiments, collection of human IVD samples, supervision of the experiments and writing of the manuscript. All authors read and approved the final manuscript.

## Supplementary Material

Additional file 1 **Subject information from cadaveric and surgical human IVD samples**. Gender, Age and Cause of Death (if applicable) is indicated for all human intervertebral disc samples used in this study. Click here for file

Additional file 2 **Primer Sequences**. Provides the sequences and Tm (°C) for the Bisulfite PCR Primers, Expression Primers, Luciferase Construct Primers and Pyrosequencing Primers used in this study. Click here for file
