# Supplementary material for: DNA methylation of SPARC and chronic low back pain
Source: Mol Pain. 2011 Aug 25;7:65. doi: 10.1186/1744-8069-7-65 (PMC3182907; doi:10.1186/1744-8069-7-65)
Supplement: Additional file 1 — Subject information from cadaveric and surgical human IVD samples. Gender, Age and Cause of Death (if applicable) is indicated for all human intervertebral disc samples used in this study. [file 1744-8069-7-65-S1.PDF]

Additional File 1: Subject information from cadaveric and surgical human IVD samples.

Subject ID	Gender	Age	Cause of Death
tq8	Male	62	Stroke (brain)
tq10	Male	62	Myocardial infarction
tq12	Male	55	Motor Vehicle accident
tq16	Male	43	Anoxia (hanging)
tq47	Male	69	Unknown
1007	Female	43	N/A
1009	Unknown	49	N/A
1015	Female	52	N/A
1016	Female	44	N/A
1106	Female	43	N/A
1112	Male	28	N/A
1124	Female	55	N/A
1172	Male	49	N/A
2000	Male	36	N/A
9634	Unknown	57	N/A
