# Supplementary material for: DNA methylation of SPARC and chronic low back pain
Source: Mol Pain. 2011 Aug 25;7:65. doi: 10.1186/1744-8069-7-65 (PMC3182907; doi:10.1186/1744-8069-7-65)
Supplement: Additional file 2 — Primer Sequences. Provides the sequences and Tm (°C) for the Bisulfite PCR Primers, Expression Primers, Luciferase Construct Primers and Pyrosequencing Primers used in this study. [file 1744-8069-7-65-S2.PDF]

Additional File 2: Primer sequences

Bisulfite PCR Primers	Sequence	Tm (^oC)
Outer Human SPARC forward	GAGGGTTATGTTTTTAAGGGGAGT	60
Outer Human SPARC reverse	CTCCAAACCTCACTTACCCTCT	60
Nested Human SPARC region 1 forward	GGGGTTGGTGTAATTATAGAAGG	60
Nested Human SPARC region 1 reverse	AAACCCAACCCAAAACTCTAAA	60
Nested Human SPARC region 2 forward	GAGTTTTGGGTTGGGTTTATTTT	60
Nested Human SPARC region 2 reverse	CATCCCTCCAAACCTATCCA	60
Outer Mouse SPARC forward	GGTTGATAGGGAGGTGTATATTT	54
Outer Mouse SPARC reverse	TCCCCCACTACCATACCAAC	54
Nested Mouse SPARC region 1 forward	AGGTAGGTAGGTAGGTAGGTAGG	60
Nested Mouse SPARC region 1 reverse	TTTTTGTTTTGTGGTAGGTTGTG	60
Nested Mouse SPARC region 2 forward	GGGTTGGAATAGTTGTTGGAA	60
Nested Mouse SPARC region 2 reverse	GAGTGAATTTGTTTGAGTATTTTT	60
**Expression Primers**		
Mouse SPARC forward	ATGAGGGCCTGGATCTTCTTTC	60
Mouse SPARC reverse	GGAAGAGTCGAAGGTCTTGTTGTC	60
Mouse GAPDH forward	GTCGTGGAGTCTACTGGTGTC	60
Mouse GAPDH reverse	GAGCCCTTCCACAATGCCAAA	60
**Luciferase Construct Primers**		
Human SPARC Sense for	AAGCTTTATAGGGGGTCACACATACCTCAG	60
Human SPARC Sense rev	GGATCCTATAAAAGAGGCTGTTCTGGGTCA	60
Human SPARC Antisense for	GGATCCTATAGGGGGTCACACATACCTCAG	60
Human SPARC Antisense rev	AAGCTTTATAAAAGAGGCTGTTCTGGGTCA	60
Mouse SPARC Sense for	AAGCTTTATACTGACAGGGAGGTGCATACC	60
Mouse SPARC Sense rev	GGATCCTATATCTGCCTTGCTGTACATTGC	60
Mouse SPARC Antisense for	GGATCCTATACTGACAGGGAGGTGCATACC	60
Mouse SPARC Antisense rev	AAGCTTTATATCTGCCTTGCTGTACATTGC	60
**Pyrosequencing Primers**		
Mouse SPARC Region 1 Sequencing 1	CCTCCACATTCCTACAACCC	N/A
Mouse SPARC Region 2 Sequencing 1	TAGGTTAGGTTTTGTTTAGAG	N/A
Mouse SPARC Region 2 Sequencing 2	GAGAGAGAGAGAGTTATAGAGGT	N/A
Human SPARC Region 1 Sequencing 1	AATTATAGAAGGGAAAGGTT	N/A
Human SPARC Region 2 Sequencing 1	AACCCTAACACTCTATAAAT	N/A
Human SPARC Region 2 Sequencing 2	TTGGGTTTATTTTTTTTTA	N/A
